# Protecting world leaders against deep fakes using facial, gestural, and vocal mannerisms

**DOI:** 10.1073/pnas.2216035119

**Published:** 2022-11-23

**Authors:** Matyáš Boháček, Hany Farid

**Affiliations:** ^a^Gymnasium of Johannes Kepler 169 00 Prague, Czech Republic; ^b^Department of Electrical and Computer Sciences, School of Information, University of California, Berkeley, CA 94708

**Keywords:** synthetic media, deep fakes, disinformation, digital forensics

## Abstract

Since their emergence a few years ago, artificial intelligence (AI)-synthesized media—so-called deep fakes—have dramatically increased in quality, sophistication, and ease of generation. Deep fakes have been weaponized for use in nonconsensual pornography, large-scale fraud, and disinformation campaigns. Of particular concern is how deep fakes will be weaponized against world leaders during election cycles or times of armed conflict. We describe an identity-based approach for protecting world leaders from deep-fake imposters. Trained on several hours of authentic video, this approach captures distinct facial, gestural, and vocal mannerisms that we show can distinguish a world leader from an impersonator or deep-fake imposter.

In the early days of the Russian invasion of Ukraine, President Zelenskyy warned the world that Russia’s digital disinformation machinery would create a deep fake of him admitting defeat and surrendering. By mid-March of 2022, a deep fake of Zelenskyy appeared with just this message (1). This video was debunked thanks to the rather crude audio and video and to Zelenskyy’s prebunking, but not before it spread across social media and appeared briefly on Ukrainian television. Three months later, the mayors of Berlin, Madrid, and Vienna each held extended video-based conversations with a deep-fake version of Kiev Mayor Klitschko.

In addition to adding jet fuel to disinformation campaigns, this new breed of synthetic media also makes it easier to deny reality—the so-called liar’s dividend (2)—as seen by the recent baseless claim that video addresses by President Biden are deep fakes deployed to conceal his death or incapacitation (3).

These recent events are likely just the beginning of a new assault on reality in both recorded and live videos. While we may have the ability to perceptually detect some deep-fake videos (4), our ability is not always reliable, and this task will become increasingly more difficult as deep fakes continue to improve in quality and sophistication.

The computational detection of deep-fake videos can be divided into three categories: 1) learning-based, in which features to distinguish the real from fake are explicitly learned (5); 2) artifact-based, in which low-level to high-level features are explicitly designed to distinguish the real from fake (6); and 3) identity-based, in which biometric-style features are used to identify whether the person depicted in a video is who it purports to be (7).

The advantage of identity-based techniques is that they are resilient to adversarial and laundering attacks and are applicable to the many different forms of deep fakes. The disadvantage is that these techniques require an identity-specific model, generated from several hours of authentic video footage. Because we are focused on protecting world leaders for whom hours of video can easily be acquired, we contend that an identity-based approach is the most sensible and robust approach.

We describe an integrated facial, gestural, and vocal model that captures an individual’s distinctive speaking mannerisms. After training a model on several hours of authentic video, the model can be used to distinguish one person from impersonators and deep-fake imposters. Given the recent attacks on President Zelenskyy—and the associated risks in the fog of war—we describe an in-depth analysis of the efficacy of this model in protecting President Zelenskyy against deep-fake attacks. Our approach, however, can be deployed to protect any world leader or other high-profile public figures.

## Results

Shown in Table 1 is the classification accuracy for distinguishing real video segments of President Zelenskyy from impersonators and deep fakes. The rows correspond to different subsets of individual (facial, gestural, or vocal) or combined features. The columns correspond to the accuracy on different datasets and different true positive rates of 95.0%, 99.0%, and 99.9% (i.e., correctly classifying a real video segment).

**Table 1.** Classification accuracy (reported as percentages) of our behavioral model trained on subsets of facial, gestural, and vocal features

																Lip-Sync					In-The-Wild		
																Deep-Fake					Deep-Fake		
Model	Real Zelenskyy					World leaders					FaceForensics++					Zelenskyy					Zelenskyy		
Facial	95.00	98.97	99.89			95.21	93.54	90.34			86.93	85.36	82.80			19.38	16.73	12.30			89.94	86.21	75.34
Gestural	94.90	98.97	99.87			69.05	61.03	52.21			66.11	61.05	55.12			15.15	8.28	1.04			30.75	24.43	18.33
Vocal	94.97	98.97	99.87			54.44	41.27	20.92			6.06	0.00	0.00			70.04	34.57	6.66			100.00	99.71	92.96
Facial + gestural	95.12	99.03	99.91			100.00	100.00	100.00			100.00	100.00	100.00			96.06	95.13	93.83			100.00	100.00	100.00
Gestural + vocal	94.96	98.99	99.91			99.09	88.86	86.37			66.17	62.01	56.97			62.32	60.56	50.39			100.00	100.00	100.00
Vocal + facial	95.01	99.05	99.91			99.85	99.75	99.29			78.06	76.59	70.75			94.43	94.20	92.95			100.00	100.00	100.00
Facial + gestural + vocal	95.19	99.05	99.89			100.00	100.00	100.00			100.00	100.00	100.00			100.00	100.00	100.00			100.00	100.00	100.00

The columns correspond to the classification accuracy for three different true positive rates (95.0% (red), 99.0% (green), and 99.9% (blue)) across real and fake (decoy and deep-fake) video segments of Zelenskyy.

With only a single feature set, the classifier struggles to consistently distinguish Zelenskyy from decoys and deep fakes. The pair-wise combined features (rows 4–6) provide a boost in accuracy with the triple features (row 7) providing a significant boost yielding perfect accuracy across all datasets. Note that the vocal features provide no benefit for the FaceForensics++ dataset because these videos contain no audio track.

**Fig. 1.**
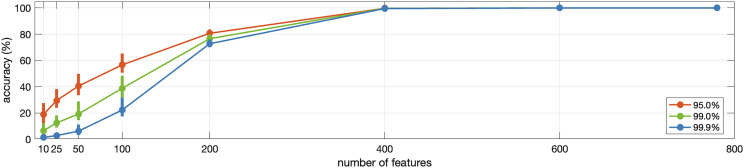
An ablation analysis in which each data point corresponds to the median (50% quantile) classification accuracy across 100 training and testing cycles using between 10 and 600 randomly selected facial, gestural, and vocal features; the error bars correspond to the 25% and 75% quantiles. The full behavioral model consists of 780 features. The three curves correspond to different true positive rates (correctly classifying a real video segment).

To determine how many of the 780 behavioral features are needed to achieve the classification accuracy reported in Table 1, we trained a series of classifiers on randomly selected subsets of features. Shown in Fig. 1 is the median accuracy of classifying the identities in the World Leaders and both Deep-Fake Zelenskyy datasets for a behavioral model with between 10 and 600 features and for three different true positive rates. Accuracy grows relatively slowly between 10 and 400 features plateauing at 99.5% (for a 99.9% true positive), increasing to 99.99% at 600 features, before topping out at 100.0% at 780 features. A significant fraction of the facial, gestural, and vocal features are informative.

We next trained 4,000 classifiers on random feature subsets of size 10. The discriminatory power of each feature is computed as the weighted average of each classifier accuracy to which a feature contributed. Classifier accuracy ranges between 18.1% and 1.2%, with a median accuracy of 2.4%. The following are five most and least discriminative features’ correlation and respective accuracy (where MFCC-*n* corresponds to vocal characteristics).

**Table:** 

Feature 1	Feature 2	Accuracy (%)
Right elbow (x)	Right shoulder (x)	18:1
Mouth (y)	AU14 (dimpler)	12:6
MFCC-1	MFCC-7	10:4
Head rotation (x-axis)	Right wrist (y)	8:0
AU12 (lip-corner pull)	AU15 (lip-corner depress)	7:9
		
Mouth (x)	AU1 (inner-brow raise)	1:3
Left wrist (y)	Right elbow (y)	1:3
AU1 (inner-brow raise)	Left wrist (x)	1:3
Right elbow (y)	MFCC-5	1:3
Right wrist (x)	MFCC-7	1:2

Note first that features from the facial, gestural, and vocal sets are each represented in the top-five most distinctive features. The most distinctive feature corresponds to the particular way that President Zelenskyy tends to gesture with his left arm while leaving his right arm dangling to his side, yielding a strong correlation between the movement of his right elbow and right shoulder as he moves side to side. The AU12-AU15 feature corresponds to a slight but consistent asymmetry in President Zelenskyy’s smile where the right side of his mouth turns slightly upward while the left side turns slightly downward. These highly specific correlations suggest that a deep-fake imposter will have considerable difficulty in precisely capturing a person’s behavioral.

## Discussion

Any approach to detecting manipulated media is vulnerable to the inherent cat-and-mouse game between the creator and detector. In our case, however, we benefit from a three-pronged approach that analyzes facial, gestural, and vocal patterns and, importantly, the interplay between them. Synthesis engines are unaware of these semantic patterns, and therefore, direct counterattack by modeling these patterns is (currently) unlikely. We also benefit from a relatively long analysis time window (10 s). By comparison, synthesis engines (currently) generate only one video frame, or a few, at a time and are therefore unaware of these longer temporal patterns. Despite this seemingly upper hand, we do not expect to publicly release our classifier so as to slow counterattacks. We will, however, make our classifier available to reputable news and government agencies working to counter deep-fake-fueled disinformation campaigns.

Although we have focused our analysis on one world leader, our approach is applicable to any world leader or high-profile individual for whom sufficient authentic video is available. Our experience has been that at least 8 h of training video is required. (By focusing on a few central repositories, this data can be efficiently scraped in a matter of hours.)

Deep fakes continue their trajectory of creating increasingly higher quality fake videos while continually lowering barriers to creation in terms of data, computing power, and technical skill required. This democratized access to powerful video synthesis technology is sure to yield exciting and entertaining applications while simultaneously posing new threats to our ability to trust what we see and hear online.

### Dataset

We downloaded 506 min of video of President Zelenskyy from YouTube and the official website of the office of the Ukrainian president* in four different contexts: a) public address (91 min); b) press briefing (207 min); c) bunker (47 min); and d) armchair (161 min). Portions of each video with large camera motions (e.g., zoom, translation, cross-fade) were automatically detected and removed from the dataset.

A total of 57 min of interview-style videos of seven world leaders (Jacinda Ardern, Joe Biden, Kamala Harris, Boris Johnson, Wladimir Klitschko, Angela Merkel, and Vladimir Putin) were used as decoys (i.e., not Zelenskyy). Our deep-fake detection is designed to distinguish Zelenskyy’s behavioral and gestural mannerisms from imposters driving the creation of a deep fake, so these decoy videos—regardless of the identities—serve as proxies for deep fakes. An additional 63 min of video from 250 distinct individuals in the FaceForensics++ FaceShifter dataset (8) was used as additional decoys. Three commissioned lip-sync deep fakes (2 min) created by the team at Colossyan† and one in-the-wild deep fake (1 min) were added to this decoy dataset. Described next are three sets of identifying features extracted from these datasets by partitioning each video into overlapping (by 1/6-s) 10-s video segments.

### Facial Mannerisms

For each video segment, the OpenFace2 toolkit (9) extracts facial landmark positions, facial action units, head pose, and eye gaze on a per-frame basis.‡ Facial muscle movement and expression are encoded using facial action units (AUs) (11). The OpenFace2 toolkit provides—on a per-frame basis—the strength of 16 different AUs. Our model incorporates these AUs and four additional features: 1) head rotation about the x-axis; 2) head rotation about the z-axis; and 3, 4) the horizontal distance between the corners of the mouth and the vertical distance between the upper and lower lip, yielding a total of 20 facial mannerism features per video segment.

### Gestural Mannerisms

For each video segment, the arm and hand positions and movement are estimated on a per-frame basis using Blazepose (12) from the MediaPipe library (13). Because we are interested only in the upper body, we consider the image *x*-, *y*-coordinates corresponding to the shoulder, elbow, and wrist of both arms, yielding a total of 12 individual measurements per video segment. These upper-body coordinates, initially specified relative to the video-frame size, are normalized into a speaker-centric action plane (14). This action plane is a rectangular bounding box centered on the speaker’s chest with a width 8× and height 6× the measured head height (15). This normalization ensures that the tracked upper-body coordinates can be compared across different speaker locations and sizes.

### Vocal Mannerisms

For each audio segment (corresponding to a video segment), the mel frequency cepstral coefficients (MFCCs) (16) are extracted. To do so, a periodogram is first estimated (using a Hann window), followed by an application of a mel-spaced filter bank and normalization by the respective filter bandwidths. Last, the MFCCs are computed using a discrete cosine transform (DCT-III) on the log-scaled weights, yielding an eight-dimensional signal per video segment.

### Behavioral Model

Correlations between all pairs of the 20 facial, 12 gestural, and 8 vocal features are used to capture individualized mannerisms. A total of _40_*C*_2_ = (40 × 39)/2 = 780 correlations are extracted from overlapping 10-s video segments extracted from an input video.

Trained on authentic video of a person of interest, a novelty detection model in the form of a one-class, nonlinear support vector machine (SVM) (17) is used to distinguish an individual from impersonators and deep fakes. The 123 min of decoy identities in the World Leaders, FaceForensics++, and Deep-Fake Zelenskyy videos are partitioned into overlapping (by 1/6-s) video segments, yielding a total of 25,267 segments. The 506 min of Zelenskyy video is similarly partitioned yielding a total of 157,746 segments. The authentic segments are then randomly partitioned into a 80/10/10 training/validation/testing split. The SVM hyperparameters, consisting of the Gaussian kernel width (*γ*) and outlier percentage (*ν*), are optimized by performing a grid search over these parameters across the training set. The validation set is used to determine the SVM classification threshold that yields a specified true positive rate (correctly classifying a video segment as Zelenskyy). The classifier is then evaluated against the testing set. This entire process is repeated 10 times with randomized data splits, from which we report average classification accuracy in Table 1 and Fig. 1.

## Data Availability

Some study data available (The data associated with this manuscripts includes training videos which we will make available. We prefer not to make available the other data in the form of the trained behavioral models because we fear that this could be used by an adversary to evaluate the realism of fake videos. We will, however, upon request make our model available to researchers working in the general space of digital forensics) (18).

## Footnotes

^*^https://www.president.gov.ua/en/videos/videos-archive.

^†^Z. Kovacs, M. S. Aslam, and the Colossyan team (https://www.colossyan.com) created the Zelenskyy deep fakes.

^‡^Two aspects of the default OpenFace2 configuration are changed to ensure more consistent facial tracking: 1) the Multi-PIE (10) model is always used and 2) the temporal smoothing of action units is disabled.
